# Perceiving object affordances through visual and linguistic pathways: A comparative study

**DOI:** 10.1038/srep26806

**Published:** 2016-05-25

**Authors:** Zuo Zhang, Yaoru Sun, Glyn W. Humphreys

**Affiliations:** 1 Department of Computer Science and Technology, Tongji University, Shanghai, P.R. China; 2 Department of Experimental Psychology, University of Oxford, Oxford, UK

## Abstract

It is known that both perceiving visual objects and reading object names automatically activate associated motor codes and modulate motor responses. We examined the nature of these motor activation effects for different effectors (hands and feet), and for pictures and words, across the time course of responding. The compatibility effects elicited by objects and words were comparable for the mean effect size, both were larger for slow than for fast responses and the effects were positively correlated across the stimulus types. Our results support an embodied cognition account in which the perception of objects and words automatically activates perceptual simulations of the associated actions, suggesting that objects and words share cognitive and neural mechanisms for accessing motor codes. However, the compatibility effects for objects and words carried over across trials differently: the compatibility effect for words was sensitive to a previous response, while the effect for objects was more immune to such influence. This result suggests a stronger link between objects and actions through a visual pathway than through a linguistic pathway.

Object affordance refers to actions that match with the physical properties of an object^1^. The embodied theory of cognition proposes that perceiving object affordance is grounded in sensorimotor systems in the brain^2^,^3^. Studies with both normal observers and brain injured patients have provided converging evidence that perceiving objects^4^,^5^,^6^,^7^,^8^,^9^,^10^,^11^ and object names^12^,^13^,^14^,^15^,^16^ activates their associated motor codes automatically and can modulate motor responses. This coupling between object perception and action can be indexed experimentally through stimulus-response compatibility effects, which reflect faster responses for actions compatible with the object affordance, relative to incompatible actions^7^,^9^,^10^,^11^. Interestingly, responses to an object were shown to be faster if the grip size (power grip or precision grip) for responding was compatible with the object’s size even under a natural/manmade discrimination task that was irrelevant to object affordance, furthermore the compatible effects elicited by object names were shown to be comparable to those elicited by the corresponding objects^5^. In the literature, both objects and words have been revealed to be capable of facilitating^5^ and interfering^17^ with motor responses in a similar fashion on the affordance processing.

However, some recent studies have also shown that objects and words actually recruit the motor system differently. Although performance on contextual decisions (e.g., used in a kitchen or not) is similar for objects and corresponding words, action decisions (e.g., twisting vs. pouring) are significantly faster for objects, suggesting a priority for objects over words in accessing associated actions^18^. Moreover, objects have been found to elicit compatibility effects within the reachable space only, while the compatibility effect between the grip used for responding and the grip evoked by the object’s name was not limited by participants’ reaching range, for judging whether an object presented either within or out of the reachable space matched with a previously displayed object name^11^. These results suggest that motor activations evoked by objects and words may be partially dissociated.

Apart from what kind of dissociation objects and words evoking motor codes still remains uncertain, until now, the clear property of the compatibility effects for objects and words has not been directly compared, including whether the compatibility effects for objects and words are correlated, whether they similarly evolve over response time, and whether they similarly carry over across trials. The compatibility effects for objects^6^,^19^,^20^,^21^ and words^12^ have been found to become larger for longer response times, but it is still unknown whether the pattern of the increasing influence on the motor system is comparable for objects and words. The carry-over effect, on the other hand, reflects how the compatibility effect is affected by previous responses, and may involve conflict adaptation^22^ and feature integration processes^23^,^24^. It has been shown that the compatibility effect for objects is reduced following an incompatible trial relative to a compatible one^10^, nevertheless, how the compatibility effect for words carries over across trials is still unclear. Of interest to the presented study here was that a strong carry-over effect would indicate that the compatibility effect is sensitive to the influence from a previous response, while a weak carry-over effect would reflect robustness of the compatibility effect to previous influences.

In this study, we assessed the motor activations elicited by objects and corresponding words by comparing various aspects of the compatibility effects. In Experiment 1, we had participants prepare hand or foot responses according to an instructional cue at the beginning of each trial and judge whether an object (or a word) was hand-related or foot-related. The response was made by either the left or the right effector (hand or foot) indicated by the cue. In this way the effector used to respond was orthogonal to the effector-related objects, giving rise to compatible and incompatible conditions. The compatibility effects for objects and words were compared on the following aspects: a) the mean effect sizes; b) how the compatibility effects evolved over response time; and c) whether the compatibility effects were correlated between the two stimulus types. In Experiment 2, participants were asked to switch between hand and foot responses after fixed blocks of trials. We examined whether the compatibility effect existed if the response effector was held in memory instead of being indicated by a trial-by-trial instructional cue. The trial sequences were carefully designed for analysing how the compatibility effect carried over across trials. The compatibility effects for objects and words were compared in order to reveal the similarities and differences in the motor activations elicited by the two stimulus types.

## Results

### Experiment 1

One participant was excluded for low response accuracies (more than 2 SDs lower than the group’s mean accuracy). Another participant was excluded for very slow response times (more than 2 SDs above the group’s mean response time). The remaining data from 30 participants were subjected to a 2 × 2 × 2 ANOVA analysis involving the following within-subjects factors: Stimulus Type (object vs. word), Stimulus Effector (foot-related vs. hand-related) and Response Effector (foot response vs. hand response).

#### Comparing compatibility effect sizes between objects and words

**Figure 1:**
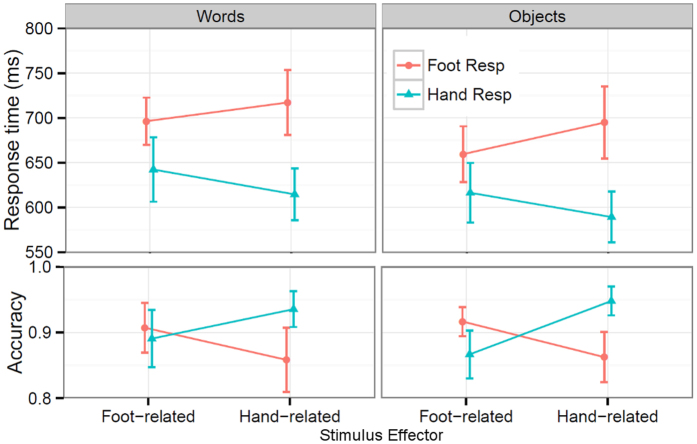
Group-level response times and accuracies (means and 95% confidence intervals^36^) for each condition.

The ANOVA on the accuracy data (Fig. 1) showed a significant effect of Response Effector (*F*(1, 29) = 6.49, *p* = 0.016), reflecting higher accuracies for hand responses (91.0%) than for foot responses (88.6%). There was a significant Response Effector × Stimulus Effector interaction, i.e. a compatibility effect (*F*(1, 29) = 26.82, *p* < 0.0001): hand responses were more accurate to hand-related stimuli than to foot-related stimuli (*F*(1, 29) = 20.78, *p* < 0.0001), and the reverse was found for foot responses (*F*(1, 29) = 10.91, *p* = 0.003).

The ANOVA on the response time (RT) data showed a significant main effect of Stimulus Type (*F*(1, 29) = 32.61, *p* < 0.0001), reflecting faster responses to objects (640 ms) than to words (667 ms). A significant main effect of Response Effector (*F*(1, 29) = 115.45, *p* < 0.0001) was found, indicating faster hand responses (616 ms) than foot responses (691 ms). There was also a significant Response Effector × Stimulus Effector interaction, i.e. a compatibility effect (*F*(1, 29) = 23.98, *p* < 0.0001): hand responses were faster to hand-related stimuli than to foot-related stimuli (*F*(1, 29) = 23.52, *p* < 0.0001), and the reverse was found for foot responses (*F*(1, 29) = 9.28, *p* = 0.005), as shown in Fig. 1. The result of a non-significant interaction of Stimulus Type × Response Effector × Stimulus Effector (*F*(1, 29) = 1.05, *p* = 0.31) showed that the compatibility effects were comparable for objects and words (29.11 ms vs. 21.83 ms).

#### How does the compatibility effect evolve over time?

**Figure 2:**
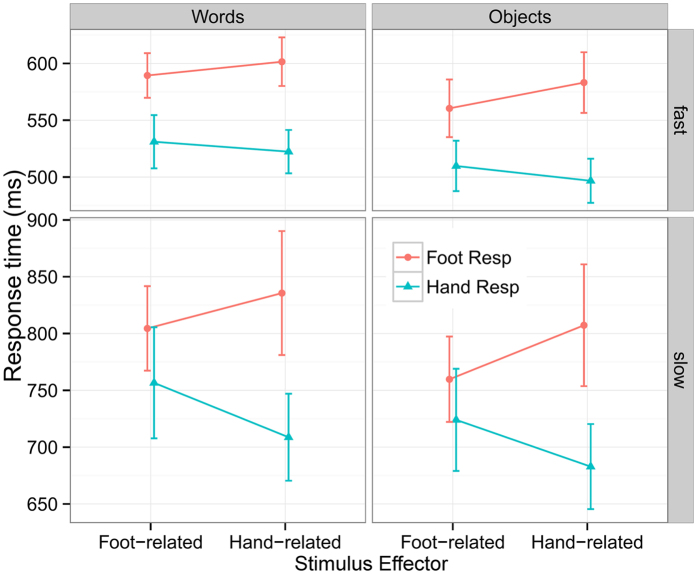
The compatibility effects for fast and slow responses. The error bars represent 95% confidence intervals for within-subject designs^36^.

We next included response speed (Speed: fast vs. slow responses) as an additional factor; of interest were interactions with the Speed factor. Response time on each trial was classified as ‘fast’ or ‘slow’ based on whether it was smaller or larger than the median RT in the corresponding condition. The RT data demonstrated a reliable 3-way interaction of Response Effector × Stimulus Effector × Speed (*F*(1, 29) = 16.66, *p* < 0.001). Simple effect analysis showed that the compatibility effects were significant for both fast responses (*F*(1, 29) = 13.95, *p* < 0.001) and slow responses (*F*(1, 29) = 23.66, *p* < 0.0001), and were significantly larger for slow responses. The 4-way interaction of Response Effector × Stimulus Effector × Speed × Stimulus Type was not significant (*F*(1, 29) = 0.06, *p* = 0.80). These results demonstrated that the compatibility effects became larger for slower responses, and this pattern was similar for objects and words (Fig. 2).

#### Are compatibility effects for objects and words correlated?

**Figure 3:**
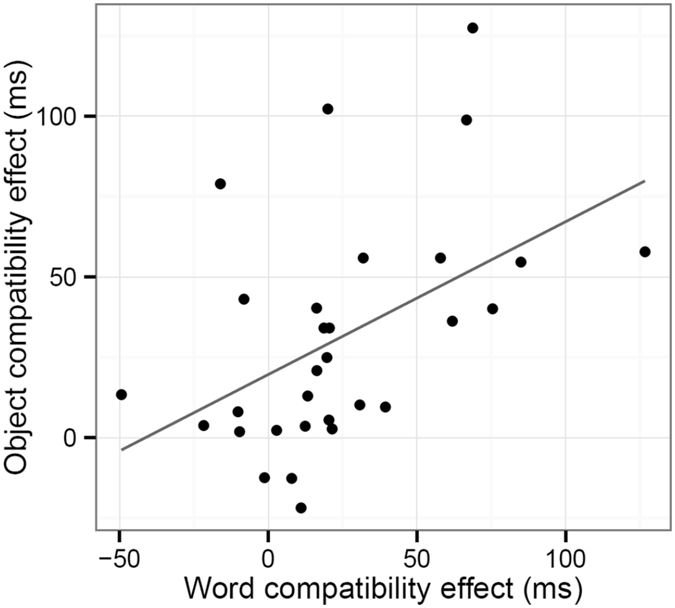
The individual compatibility effects. A significant correlation was found for the compatibility effects between objects and words.

To the compatibility effects for objects and words for each participant (Fig. 3), a significant correlation was found (Pearson’s *r*(28) = 0.48, *t* = 2.89, *p* = 0.0074).

In Experiment 1 we found significant compatibility effects, which provided evidence for the involvement of the motor system in perceiving object affordance^4^,^5^,^6^,^7^,^8^. The compability effects for objects and words were similar in the effect size, both increased over time, and were strongly correlated, suggesting that the two stimulus types elicited motor activations through common mechanisms^5^. The compatibility effects becoming larger for slower responses indicated the emergence of object-induced motor activations over time^6^,^19^,^20^,^21^.

In Experiment 2, the required response effector was blocked rather than cued on a trial-by-trial basis used in Experiment 1, in order to remove hand-foot switching from the investigation of the carry-over effect. In this way, we could examine the compatibility effects for objects and words under conditions where there was no visual cue before each trial. Could compatibility effects arise if the response effector was retained in memory? How were the compatibility effects affected by a previous response?

### Experiment 2

In Experiment 2, participants alternated between hand and foot responses every 4 trials (a “miniblock”), in order to compare how the response compatibility effect carried over across trials within the same response effector and also to minimise the influence of hand-foot switching. A cue was displayed at the start of each miniblock to indicate the response effector. Then, from trial 2 to trial 4 within each miniblock, the response effector was maintained in memory instead of being indicated by the instructional cue. One participant was excluded for low accuracies (56.2%). Another participant was excluded for very slow responses (more than 2 SDs larger than the group mean). The remaining data from 23 participants were subjected to the analyses.

### Do carry-over effects emerge for objects and words?

In order to assess the carry-over effect of compatibility, we focused on trials 2 to 4 that were free from the influence of hand-foot switching and the instructional cue. Response times and accuracies were subjected to within-subject ANOVAs involving Stimulus Type, Compatibility and PreCompatibility (compatibility on a previous trial: compatible vs. incompatible) as factors.

**Figure 4:**
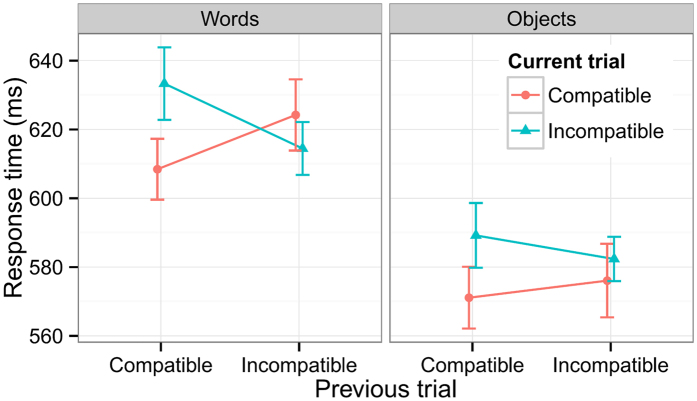
The carry-over effect of compatibility for objects and words.

The ANOVA on accuracies showed a single significant effect of Stimulus Type (*F*(1, 22) = 24.96, *p* < 0.0001), reflecting more accurate responses to objects (95.8%) than to words (92.9%). For the RT data there were significant main effects of Stimulus Type (*F*(1, 22) = 45.32, *p* < 0.0001) and Compatibility (*F*(1, 22) = 12.32, *p* = 0.002). The Compatibility × Stimulus Type interaction was not significant (*F*(1, 22) = 1.86, *p* = 0.19), indicating that the compatibility effects for objects and words were comparable (10.96 ms vs. 10.90 ms). Of most interest was a significant carry-over effect of compatibility (Compatibility × PreCompatibility, *F*(1, 22) = 14.90, *p* < 0.001), reflecting stronger compatibility effects following a compatible trial (*F*(1, 22) = 17.87, *p* < 0.001) than following an incompatible one (*F*(1, 22) = 0.37, *p* = 0.55). This in turn interacted significantly with Stimulus Type (*F*(1, 22) = 6.47, *p* = 0.019, Fig. 4). Simple effect analysis showed that Compatibility × PreCompatibility was significant for words (*F*(1, 22) = 22.52, *p* < 0.0001), but not for objects (*F*(1, 22) = 2.34, *p* = 0.14). For words, the compatibility effect was significant following a compatible trial (*F*(1, 22) = 24.80, *p* < 0.0001), but a reversed compatibility effect was found following an incompatible trial (*F*(1, 22) = 4.42, *p* = 0.047). These results demonstrated that the motor activation induced by words was sensitive to a previous response. In contrast, the motor activation induced by viewing objects was relatively immune to the influence from a previous trial.

## Discussion

In this study, we compared how viewing objects and reading words activated associated actions by examining various aspects of the compatibility effects. Both Experiment 1 and 2 found that the compatibility effect sizes were comparable for the two stimulus types. Experiment 1 also revealed that the compatibility effects for objects and words both became larger for longer RTs, and were strongly correlated. Experiment 2 focused mainly on the carry-over effect from previous trials and its results demonstrated that the compatibility effect for objects was less affected by previous responses than that for words.

### Similarities for objects and words

We assessed the compatibility effects for objects and words and found results consistent with previous findings in that both objects and words significantly modulated motor responses^5^,^17^. These results support the embodied cognition accounts which hold that access to motor codes is an integral part of object perception^2^,^3^. The comparable compatibility effects for objects and words were demonstrated not only for the mean effect size, but also for the detailed characteristics of the effects: the evolution of the effects over time and the strong correlation across the stimulus types. These results consolidate prior propositions that perceiving objects activates associated motor codes regardless of the modality of the perceptual input^5^, suggesting that shared motor activations are automatically elicited by perceiving objects and their corresponding words.

Our result of the compatibility effects increasing for the longer RTs fits with previous results on how object-based compatibility effects distribute over RTs^6^,^12^,^19^,^20^,^21^, but previous studies have not provided a clear explanation for this finding. This suggests that, on trials with slower RTs, there is more time for the alternative response (e.g., the hand response to a hand object, where a foot response is required) to be activated and to compete for action. This result can be further explained in a double-route framework^25^: a fast dorsal neural route that is motoric in nature and a slower ventral neural route that is semantic. The compatibility effect becoming larger for longer RTs may reflect the contribution of these two processing routes at different timings. The compatibility effect for the fast responses reflected motor activation mainly through the fast dorsal route, while the compatibility effect for the slow responses reflected the combined motor activation through both the dorsal and ventral routes. Critically, we found that the compatibility effect induced by words increased over time^12^ in a manner similar to that found with objects. These data suggest that words and objects share certain cognitive and neural mechanisms for activating associated motor codes.

### Carry-over effect

The carry-over effect of compatibility was consistent with the previous findings involving object-based compatibility effects^10^ and other stimulus-response compatibility paradigms, such as the Eriksen flanker task^26^, Stroop task^27^ and Simon task^28^. A conflict adaptation account^22^ for the carry-over effect of compatibility proposed that the object-induced motor activations needed to be suppressed on incompatible trials to make correct responses, while on compatible trials such cognitive control was not required. The enhanced cognitive control required by incompatible trials carried over to the following trial, on which the object-induced motor activations were effectively suppressed and thus gave rise to reduced compatibility effects.

The conflict adaptation account predicted reduced or at best diminished compatibility effects after incompatible trials. On the trials involving words, however, the compatibility effects following incompatible trials were reversed in Experiment 2. The reversal of the compatibility effect after incompatible trials was also observed in previous studies and was attributed to the repetition priming effects^29^,^30^. A feature-integration account^23^,^24^ attributes the carry-over effect of compatibility to the repetition priming effects rather than the cognitive control mechanisms: the stimulus-response mapping on the previous trial remains in the episodic memory, and performance on the current trial is faster if the same stimulus-response mapping occurs. It should be noted that conflict adaptation and repetition priming are not mutually exclusive, and they can contribute to the carry-over effect simoutaneously^31^.

In addition, response repetition could also contribute to the carry-over effect in our data. Response repetition induced motor adaptation^32^, making a repeated response faster than a switched one. Following an incompatible response, another incompatible response involved a response repetition, while a compatible response involved a left-right response switch. Therefore, the compatibility effect was reduced due to response repetition following an incompatible response.

### Differences for objects and words

We presented a novel result that, although indistinguishable in the compatibility effect itself, the carry-over effect showed significant difference for objects and words: the compatibility effect for words was strongly affected by a previous response, while the compatibility effect for objects was insensitive to such influences. This result could be explained in light of the work by Humphreys and colleagues^18^,^33^, who have argued that the associated actions are more readily activated through a visual pathway than through a linguistic pathway. Therefore, the motor activations caused by objects are more robust and thus more immune to influences from previous responses. This visual pathway advantage could be attributed to the visual properties that are readily associated with actions, shaped and strengthened by interacting with objects in the everyday life. Words, however, although elicit embodied motor simulations, are devoid of the direct mapping between vision and action.

The difference between objects and words on the carry-over effect may suggest their partially different neural processes, namely, the affordance processing for objects and words may involve both a dorsal and a ventral neural route^25^, but to different degrees. The dorsal route is fast and motoric, and can activate motor codes more quickly and strongly than the ventral route that is semantic and slower. The result that objects were processed faster than words (a main effect of Stimulus Type) in our data suggests that objects may involve the dorsal route more than words do. Motor adaptation induced by response repetition within a miniblock is more likely to attenuate the effects generated by the fast dorsal stream and this may have different effects for the motor activation elicited by objects and words. For objects, the motor activation trough the dorsal stream is strong and is only partly cancelled by motor adaptation, reflected in a weak carry-over effect. In contrast, the motor activation induced by words through the dorsal stream is weak and might be substantially cancelled by motor adaptation, reflected in a significant carry-over effect, and especially reflected in a reversed compatibility effect following an incompatible response.

## Methods

### Experiment 1

#### Participants

Thirty-two healthy participants (age mean = 23.0, SD = 3.9, 23 females, two left-handed) volunteered to take part in this study. They were all native English speakers with normal or corrected-normal vision. All the participants gave informed consent and were paid for their participation. The experimental procedure was approved by a local Oxford University ethics committee and was carried out in accordance with the approved guidelines.

#### Experimental design and procedure

All the stimuli were presented on the centre of a computer screen (23” LCD, 100 Hz refresh rate). The eye-screen distance was approximately 60 cm and participants viewed a cue “H” or “F” followed by an object picture or a word. The task was to judge whether the stimulus was hand-related or foot-related. There were 4 responses to choose from: left/right × hand/foot responses, corresponding to two keyboard buttons (“z” and “m”) and two foot pedals. The “H” cue indicated that this trial required hand responses and “F” indicated foot responses. For half of the participants hand-related objects required left responses (either by the left hand or the left foot, depending on the cue), and foot-related objects required right responses. For the other participants this mapping was reversed. There were 3 within-subject factors: Stimulus Type, Stimulus Effector and Response Effector.

**Figure 5:**
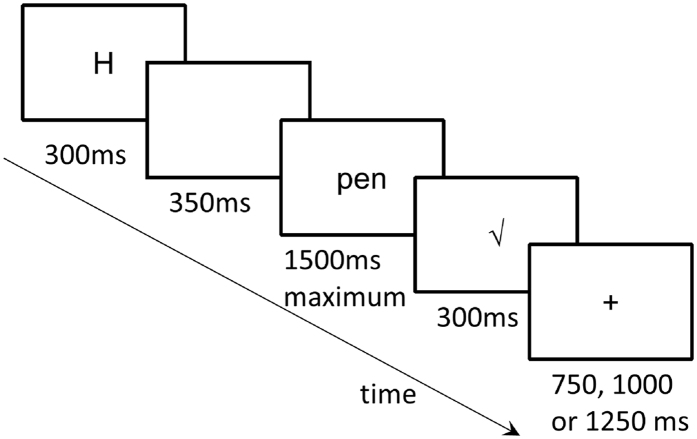
Trial procedure in Experiment 1.

Each trial started with a cue “H” or “F” (0.48° × 0.76° of visual angles) displayed for 300 ms, followed by a blank screen for 350 ms, then by an object picture (4.05° × 4.05°) or a word (height = 0.95°, width = 1.53° ~ 4.48°) for a maximum of 1500 ms. Participants were asked to respond as quickly and accurately as possible. The stimulus disappeared upon a response and was replaced with a visual feedback: “√”, “ × ”, or “too slow!” if no response was made within the 1500 ms slot. The inter trial interval was randomised between 750 ms, 1000 ms and 1250 ms (Fig. 5). E-prime software (version 2.0) was used to run the experiment.

The participants finished 1 practice block followed by 4 experimental blocks, with self-paced breaks in between. The practice block consisted of 64 trials, and the objects used in the practice block were different from those used in the experimental blocks. The first half of the practice block involved one stimulus type (objects or words) and the second half involved the other type, with counterbalanced orders. Each experimental block consisted of 64 trials and involved only one stimulus type. Blocks involving objects and words were alternated. Objects were separated into 2 subgroups, as were the corresponding words. One subgroup of the stimuli was presented in each block. The order of the experimental blocks was counterbalanced between the participants.

## Materials

Sixteen object pictures and the corresponding words (Supplementary Table S1) were selected from the BOSS dataset^34^,^35^ and used in the experimental blocks. Eight of the objects were dominantly associated with hand actions, the other eight with foot actions, judged by an independent group of 8 healthy native English speakers (5 males, aged 18 ~ 42). Four of the participants were asked to categorise each of the pictures as either hand-related or foot-related, and the other 4 judged the words. The effector-association of each stimulus was consistent across all the participants.

Some hand-related objects in the pictures (the marker, the pen and the scissors) were rotated to a vertical orientation so that they did not preferably afford left or right hand actions. Measures of the familiarity and visual complexity for the objects were obtained from the BOSS dataset^34^,^35^ and were matched between the hand-related and foot-related objects. Word lengths and frequencies were matched between the hand-related and foot-related words (Table 1). Word frequencies were obtained from the WebCelex database (Max Planck Institute for Psycholinguistics, http://celex.mpi.nl).

### Data analysis

Incorrect responses were excluded from the RT analysis. Response times beyond 3 SDs from the mean for each participant were also excluded (1.6% of corrected responses). The within-subject confidence intervals in the graphs were adjusted by Morey’s method^36^ to account for between-subject variability. For the first part of our analysis, a within-subject ANOVA was performed on RTs and accuracies with Stimulus Type, Response Effector and Stimulus Effector as factors. Secondly, response speed (Speed) was introduced as a within-subject factor, to assess the compatibility effects for fast and slow responses. A median RT was obtained for each condition of each participant. The response time on each trial was categorised as “fast” or “slow” depending on whether it was smaller or larger than the median RT. A response was excluded if it was equal to the median. Thirdly, to assess the association between the compatibility effects for objects and words, individual compatibility effects were calculated and subjected to a corrrelation analysis across the stimulus types.

### Experiment 2

#### Participants

Twenty-five healthy native English speakers participated in this experiment (19 females, age mean = 24.7, age SD = 5.3). They were all right-handed, and had normal or corrected-to-normal vision. All the participants signed informed consent and were paid for the participation. None of the participants took part in Experiment 1.

#### Experimental design and procedure

**Figure 6:**
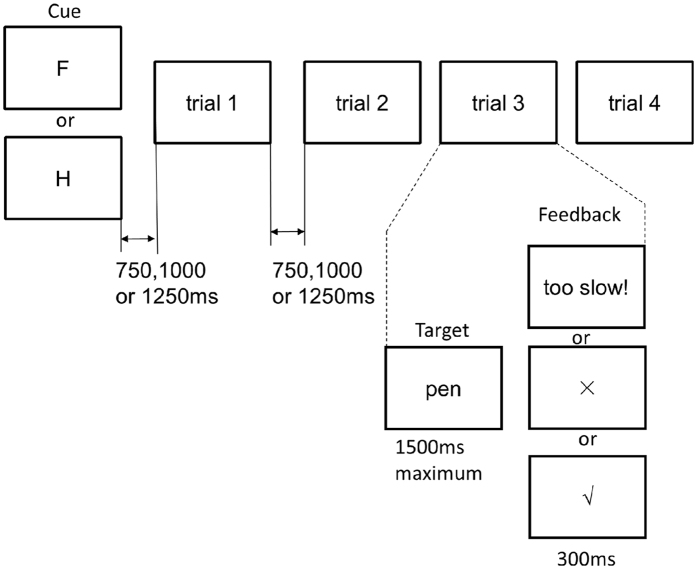
The procedure within a miniblock in Experiment 2.

The task and the stimuli were the same as in Experiment 1. Hand and foot responses were grouped into “miniblocks” (Fig. 6). Each cue and the following 4 trials formed a miniblock, in which participants responded with either the hands or the feet, so that there were no switches between the response effectors. Miniblocks cued by “H” and “F” alternated with each other. Each trial within a miniblock could be a compatible or an incompatible trial, yielding 16 different sequences of the 4 trials (2^4^ = 16). Each sequence appeared once for both the “H”-cued and the “F”-cued miniblocks, yielding 32 miniblocks, which formed an overall block. There were 1 practice block (32 trials) and 8 experimental blocks (128 trials in each block), with self-paced breaks in between. Only one stimulus type was used in each experimental block. Word blocks and object blocks alternated with each other. Which stimulus type (objects or words) was used in the first block and which side (left or right) was mapped to hand-related stimuli was counterbalanced between participants. The procedure of each trial was the same as in Experiment 1, except that no instructional cues preceded trials 2 to 4.

#### Data analysis

For the RT analysis, incorrect responses were excluded. For trials 2 to 4 within each miniblock, a trial was also excluded if the previous response was incorrect. For the remaining data, RTs beyond 3SDs from each individual’s mean were excluded (2.0%). Data on trials 2 to 4 were involved in a within-subject ANOVA with the factors being Stimulus Type, Compatibility and PreCompatibility.

## Additional Information

**How to cite this article**: Zhang, Z. *et al*. Perceiving object affordances through visual and linguistic pathways: A comparative study. *Sci. Rep.* **6**, 26806; doi: 10.1038/srep26806 (2016).

## Supplementary Material

Supplementary Information

## Figures and Tables

**Table 1:** Attributes of the stimuli (means with SDs in parentheses).

**Objects**		
Effector-association	Familiarity	Visual complexity
Foot-related	4.25 (0.68)	2.26 (0.45)
Hand-related	4.42 (0.29)	2.31 (0.49)
P value	0.52	0.81
Words		
Effector-association	Length	Frequency (per million)
Foot-related	6.13 (2.90)	3.88 (5.03)
Hand-related	7.00 (2.33)	6.63 (6.63)
P value	0.51	0.37

P values were obtained from two-sample *t*-tests between the hand-related and foot-related stimuli.
