# Supplementary material for: Perceiving object affordances through visual and linguistic pathways: A comparative study
Source: Sci Rep. 2016 May 25;6:26806. doi: 10.1038/srep26806 (PMC4879702; doi:10.1038/srep26806)
Supplement: Supplementary Information [file srep26806-s1.doc]

**Supplementary information**

Perceiving object affordances through visual and linguistic pathways: A comparative study

Zuo Zhang^1, 2^, Yaoru Sun^1*^ and Glyn W. Humphreys^2*^

1 Department of Computer Science and Technology, Tongji University, Shanghai, P.R. China

2 Department of Experimental Psychology, University of Oxford, Oxford, UK

*Correspondences to: Prof. Yaoru Sun (yaoru@tongji.edu.cn) or Prof. Glyn W. Humphreys (glyn.humphreys@psy.ox.ac.uk).

Supplementary Table S1 Attributes of objects and words used in the experimental blocks

| Object name | Related effector | Object familiarity | Visual complexity | Word length | Word frequency (per million) |
| --- | --- | --- | --- | --- | --- |
| pen | hand | 3.95 | 2.13 | 3 | 19 |
| scissors | hand | 4.08 | 2.51 | 8 | 4 |
| lighter | hand | 4.31 | 2.69 | 7 | 3 |
| guitar | hand | 4.52 | 2.45 | 6 | 6 |
| marker | hand | 4.54 | 2.13 | 6 | 2 |
| calculator | hand | 4.56 | 3.13 | 10 | 2 |
| button | hand | 4.62 | 1.62 | 6 | 15 |
| basketball | hand | 4.79 | 1.83 | 10 | 2 |
| boot | foot | 2.77 | 2.29 | 4 | 9 |
| shoe | foot | 3.79 | 2.38 | 4 | 14 |
| skis | foot | 4.17 | 2.05 | 4 | 3 |
| skateboard | foot | 4.52 | 1.88 | 10 | 0 |
| rollerblade | foot | 4.57 | 2.74 | 11 | 0 |
| sock | foot | 4.64 | 1.6 | 4 | 3 |
| skate | foot | 4.74 | 2.97 | 5 | 1 |
| slipper | foot | 4.79 | 2.13 | 7 | 1 |

Note: Object familiarities and visual complexities were obtained from the BOSS dataset^1,2^. Word frequencies were obtained from the WebCelex database (Max Planck Institute for Psycholinguistics, http://celex.mpi.nl).

# Supplementary references

1. Brodeur, M. B., Guérard, K. & Bouras, M. Bank of standardized stimuli (BOSS) phase II: 930 new normative photos. *PLoS ONE* 9, e106953 (2014).

2. Brodeur, M. B., Dionne-Dostie, E., Montreuil, T. & Lepage, M. The bank of standardized stimuli (BOSS), a new set of 480 normative photos of objects to be used as visual stimuli in cognitive research. *PLoS ONE* 5, e10773 (2010).
